# PromBase: a web resource for various genomic features and predicted promoters in prokaryotic genomes

**DOI:** 10.1186/1756-0500-4-257

**Published:** 2011-07-22

**Authors:** Vetriselvi Rangannan, Manju Bansal

**Affiliations:** 1 Molecular Biophysics Unit, Indian Institute of Science, Bangalore-560 012, India

## Abstract

**Background:**

As more and more genomes are being sequenced, an overview of their genomic features and annotation of their functional elements, which control the expression of each gene or transcription unit of the genome, is a fundamental challenge in genomics and bioinformatics.

**Findings:**

Relative stability of DNA sequence has been used to predict promoter regions in 913 microbial genomic sequences with GC-content ranging from 16.6% to 74.9%. Irrespective of the genome GC-content the relative stability based promoter prediction method has already been proven to be robust in terms of recall and precision. The predicted promoter regions for the 913 microbial genomes have been accumulated in a database called PromBase. Promoter search can be carried out in PromBase either by specifying the gene name or the genomic position. Each predicted promoter region has been assigned to a reliability class (low, medium, high, very high and highest) based on the difference between its average free energy and the downstream region. The recall and precision values for each class are shown graphically in PromBase. In addition, PromBase provides detailed information about base composition, CDS and CG/TA skews for each genome and various DNA sequence dependent structural properties (average free energy, curvature and bendability) in the vicinity of all annotated translation start sites (TLS).

**Conclusion:**

PromBase is a database, which contains predicted promoter regions and detailed analysis of various genomic features for 913 microbial genomes. PromBase can serve as a valuable resource for comparative genomics study and help the experimentalist to rapidly access detailed information on various genomic features and putative promoter regions in any given genome. This database is freely accessible for academic and non- academic users via the worldwide web http://nucleix.mbu.iisc.ernet.in/prombase/.

## Introduction

Controlling gene expression is the central process in all cellular processes. The synchronized control of gene expression is accomplished by the interplay of multiple regulatory mechanisms. Promoter elements are the key regulatory regions, which recruit the transcriptional machinery through the binding of a variety of regulatory proteins to the short oligonucleotide sequences occurring within them. Since these transcriptional regulatory elements are often short and degenerate, their identification in bacterial genomes is a difficult problem. As a consequence of large-scale genome sequencing methods and high throughput technologies, vast amount of DNA sequence data has accumulated within last decade [1]. Hence, it is essential to have highly reliable rapid annotation of functional elements, especially those responsible for controlling gene expression in organisms, since there has been only limited experimental investigation. The traditional genetic, biochemical techniques available to identify and characterize promoter regions are not readily scalable to probe whole genomes and cannot meet the challenge of the genomic era.

There are few model organisms which have been systematically annotated for promoter regions and for regulatory binding sites and curated into public domain databases. RegulonDB, Ecocyc and PromEC are the genome specific resources for *E. coli*, while DBTBS and MtbRegList provide information about *B. subtilis *and *M. tuberculosis *genomes respectively [2-6]. Recently, whole genome expression profiles have led to characterization of bacterial and archaeal transcriptomes [7-10]. Apart from the genome specific databases mentioned above, several databases involve human expertise to handle the annotations and summarize subsets of data related to different aspects of bacterial regulation. PRODORIC database provides information about operon, promoter structures, transcription factor binding sites and their position weight matrix (PWM) in prokaryotes with focus on pathogenic organisms which were collected and screened manually from the original scientific literature [11]. Tractor_DB contains a collection of computationally predicted transcription factor binding sites in gamma-proteobacterial genomes [12]. RegTransBase is a manually curated database of regulatory interactions in prokaryotes, which contains data on the regulation of about 39041 genes in 531 organisms [13]. SwissRegulon is a database containing genome-wide annotations of regulatory sites produced using multiple alignments of orthologous intergenic regions from related genomes and known sites from the literature, and ChIP-on-chip binding data [14]. However, with the increase in the number of newly sequenced genomes, it is difficult to manually curate the functional elements for them, especially for the organisms that have not been studied in detail experimentally.

The available curated databases of transcriptional regulatory regions have been extensively used to train most of the well known promoter and DNA binding site prediction algorithms developed based on sequence motifs [15-24] as well as those using structure based properties of DNA [25-30]. There are also several other databases and servers which contain computationally derived information about distribution of transcription factors in bacterial genomes [31-33]. However, none of these databases cover the entire taxonomic diversity of prokaryotic genomes and the predictions have not been validated on a genomic scale, nor do they identify promoter regions for RNA genes. Hence, this remains an important lacuna for genomic and proteomic research in microbiology.

Here we describe PromBase, a web resource that has been constructed to provide the prediction and evaluation of promoter regions in a coherent manner, so that the user can browse and search each entry or download all the predicted promoter regions for any microbial genome. Apart from being a database for putative promoter regions, PromBase provides extensive information related to other genomic features such as base composition in various intergenic and coding regions, CDS-skew and CG/TA skew along the genomic length, as well as DNA sequence dependent structural properties such as stability, curvature and bendability in the vicinity of promoter regions (-500 to +500 w.r.t TLSs). Stability profile can also be viewed for a 1001 nt spanning region with respect to TLS of each individual gene displayed in the genome browser, along with the predicted promoter regions. Hence, this database can serve as an important resource for the molecular biology community to access genome related information and facilitate planning of experiments for reliable promoter regions.

### Database content

The genome sequence for all microbial genomes was downloaded from NCBI ftp://ftp.ncbi.nih.gov/genomes/Bacteria/. Lower relative stability of DNA sequence has been used to predict promoter regions [34,35]. The method has been incorporated into an algorithm called 'PromPredict' and generalized to predict putative promoter regions in any given nucleotide sequence with a minimum length of 1000 nt. A detailed analysis of the 913 bacterial genomes, carried out after rationalizing the threshold values for identifying promoter regions in DNA sequences with varying GC-content and assigning reliability scores to the predictions, has been recently reported [36]. A standalone version of PromPredict was used to develop 'PromBase', the database presented here, which displays the predicted promoter regions, along with their evaluation parameters, in a coherent manner, so that the users can browse and search each entry or download all the predicted promoter regions for any microbial genome. The average free energy profile for a 1001 nt length sequence (spanning -500 to +500 w.r.t TLS) is also accessible for each gene displayed within the chosen variable size window. Download option is available for the predicted promoter data for all 913 microbial genomes.

In addition to acting as a resource for promoter annotation, PromBase also provides graphical representation of several other microbial genomic features such as

i. The GC-content distribution for all 1000 nt long fragments (with 250 nt overlap) in the genome along with their average free energy profiles.

ii. The cumulative CDS-skew (CDS-skew^c^) [37] as well as CG and TA skews (skew^c^) [38] for each genome.

iii. Percentage distribution, length and GC-content of different intergenic regions (tandem, divergent, convergent) as well as the protein and RNA coding regions, in each of the microbial genomes.

iv. Analysis of nucleotide composition and structural properties in promoter regions of protein coding genes

a. Distribution of A, T, G, C nucleotides in the 101 nt long sequences (-80 to +20 w.r.t TLS) and % occurrence of tetranucleotides in the vicinity of TLS (-150 to +50 versus +200 to +400 nt region w.r.t TLS).

b. %GC and average free energy distribution for different regions (-300 to -200, -80 to +20 and +200 to +300) in the vicinity of TLS.

c. CG and TA skews [39,40] for 1001 nt long sequences, spanning -500 to +500 w.r.t TLS.

d. Average free energy profile for 1001 nt long sequences in the genome (-500 to +500 w.r.t TLS and all 1001 nt long fragments with 250 nt overlap).

e. Average curvature profile using dinucleotide parameters from crystal structure (CS Model [41]) and gel mobility (BHMT model [42]) data, for the 1001 nt long sequences spanning TLSs.

f. Average bendability profile using DNase I sensitivity [43] and nucleosomal positioning preference [44] trinucleotide models, for the 1001 nt long sequences spanning TLSs.

g. Z score plot for the DNA sequence dependent structural properties such as stability, bendability and curvature (only for CS model), for the 1001 nt long sequences spanning TLSs.

**Figure 1:**
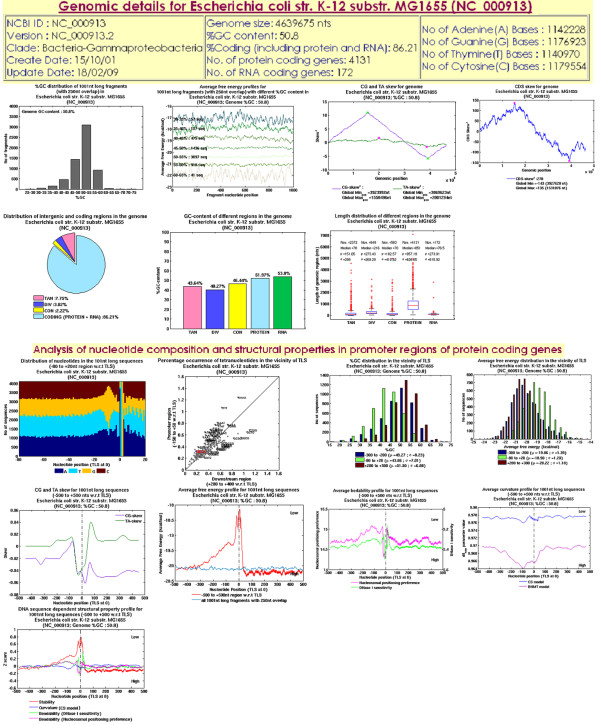
**The results provided on the web page in PromBase from analysis of genomic features for each bacterial genome**. In this figure, *E. coli K12 MG1655 *strain has been chosen as a specific example. The table at top provides the statistics of the genome. Analysis results for each feature as listed in Database content have been illustrated using a plot or histogram.

The analysis result for each feature listed above is shown in Figure 1 for *E. coli K12 MG1655 *strain. Details about the methods followed to calculate each of the above features have been provided in additional file 1.

### Database construction

The data is organized into PromBase using MySQL, a relational database management system that serves as the backend for storing data. The genome sequence data obtained from NCBI has been processed and stored into a table. The gene information for each genome and the detailed promoter prediction results for each genome are maintained in different tables. The calculated average free energy profile for the 1001 nt length sequence in the vicinity of TLS of each gene (-500 to +500 nt region w.r.t TLS) is loaded into separate table per genome. These tasks were performed with variety of SQL queries embedded in PERL scripts. The relational database schema followed for PromBase query retrieval management is summarized in Additional File 2: Figure S1. Each table has a primary key entry and the reference to them is processed internally by CGI scripts for the web interface. The fields emphasized in italic bold face font in those tables (Additional File 2: Figure S1) were used as search keys in the web interface. The figures for genomic feature analysis of each genome have been generated using scripts written in MATLAB, which is a high-level technical computing language for algorithm development, data visualization, data analysis, and numeric computation. The genome browser view in the prediction result page as well as the average free energy profiles for each gene was generated using PERL GD package. The web interface for PromBase is managed by a collection of HTML, cgi PERL scripts that do all the work, from querying the database upon user's request, to generating the dynamic web pages that form the interface. Apache is used as the web server.

## Utility

### Database interface

**Figure 2:**
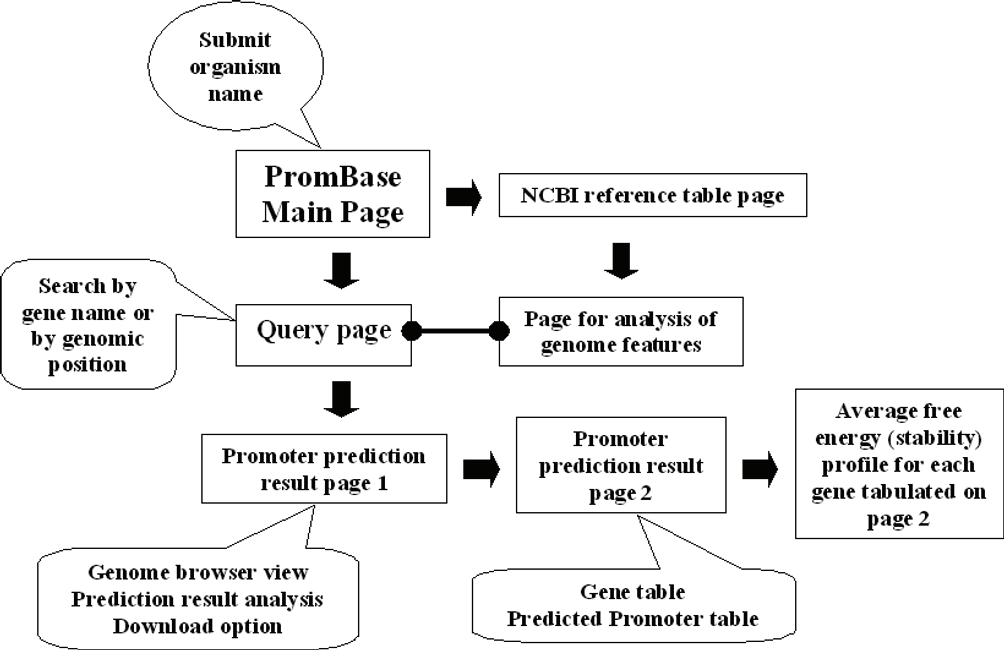
**PromBase web interface**. Rectangular box indicates a web page. Block arrows indicate the page transition caused by an action. Callouts give a simple explanation for information content availability at each page. Round headed line shows interlock between the information availability.

PromBase interface is well organized and managed at following levels. (a) Table of genome associated facts (NCBI reference table), which includes NCBI accession number, organism name with strain, size of the genome, GC composition, percentage of coding region and number of genes along with the gene product information, which were retrieved from NCBI for all 913 bacterial genomes. (b) Analysis of each genome for various features at different genomic regions as well as for sequence, and sequence dependent structural properties of DNA (as listed in previous section). (c) Detailed search results for predicted promoter regions, along with gene information, within a variable size window selected by the user, which is displayed in genome browser view. The database also correlates the predicted promoter regions with gene information in terms of true positive and false positive, depending on their location and the extensive analysis results are represented pictorially for each genome. (d) Tabulation of promoter prediction results along with the gene table that lies within the variable size window. (e) Average free energy profile for a region spanning -500 to +500 w.r.t TLS of each gene that is tabulated. Figure 2 illustrates the web interface maintained for PromBase.

### Query and data retrieval hierarchy

**Figure 3:**
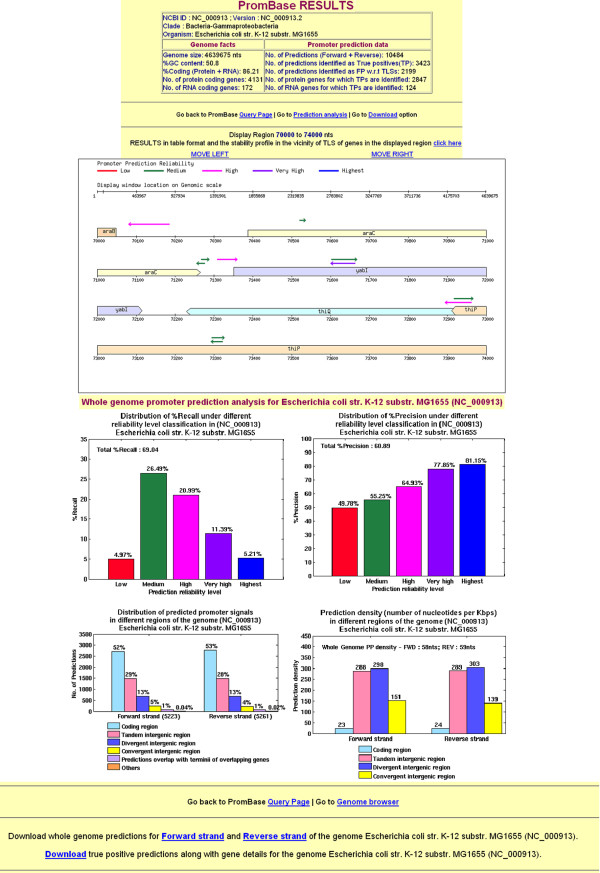
**PromBase result page for prediction and analysis of promoter regions in *E. coli K12 MG1655 *strain**. Table at top provides the statistics for the whole genome promoter prediction for *E. coli*. Genome browser view is shown for a position specific search centered on 72 Kbp of the genome with a flanking region of 2 Kbp. Histogram below illustrates the analysis of the prediction results in terms of %recall and %precision, as well as prediction distribution and density within various intergenic and coding regions.

At the first stage, an organism name is the initial query to PromBase. Upon the submission of organism name, PromBase web interface leads to a second stage query page. This page requests input for either gene name specific search or position specific search within a genomic region. The second stage query page also contains the genome feature analysis results for the query genome (Figure 1). The predicted promoter regions, along with gene information found within a variable size window selected by the user, are displayed in a genome browser view in the third stage interface (Figure 3). The exact position, nucleotide sequence and associated gene ID for the predicted promoters are also given in the tabular form in fourth stage interface (Additional File 2: Figure S2). A search using the GenBank gene ID is available at fourth stage interface, for viewing the average free energy (AFE) profile and predicted promoter region in the 500 nt flanking region, with respect to the translation start site (TLS) of the genes displayed in the genome browser view (Additional File 2: Figure S2). In future, other DNA sequence dependent structural property (such as curvature and bendability) profiles in the vicinity of TLS of each gene will also be included in PromBase.

## Discussion

### Promoter annotation in PromBase

As compared to the other databases available for prediction of regulatory elements (generally TFBS) in genomic sequences (discussed in introduction section), PromBase provides a large scale annotation of promoter regions in diverse prokaryotic genomes. Also, the predicted promoter regions have been classified into five different reliability levels (low, medium, high, very high and highest) based on the difference in their relative average free energy [36] and highlighted in PromBase using different colors. Using this reliability level classification scheme, users can design experiments with more confidence for the predictions with higher reliability levels. Within a predicted promoter region, the position corresponding to maximum difference in relative stability (DE_max_) has been highlighted, which can act as a reference position (if a predicted region is very long) for designing primers. Whole genome annotation for promoter regions and their quality, as well as distribution among various genomic regions has been analyzed extensively in terms of %recall and %precision [36] and the results are represented graphically in PromBase for each organism. The number of RNA genes with identified promoter regions has also been highlighted.

### Genome feature analysis in PromBase

In addition to being a database for promoter prediction, PromBase also analyses and shows various other genomic features (listed in the Database content section). The sequence dependent DNA structural properties (stability, bendability and curvature), CG-skew and TA-skew which have been reported to have characteristic features in the vicinity of transcription start sites (TSS) [26,37,39,45] have also been represented as plots with reference to translation start sites (TLS) for all microbial genomes. Since the TSS data is available only for a few genomes and the distance between TSS and TLS is generally small in prokaryotes, the prominent features can also be seen in a plot with respect to TLS. Comparison of the DNA sequence dependent structural property profiles in three microbial genomes (*E. coli, B. subtilis *and *M. tuberculosis*) with varying %GC-content (50.8, 43.4 and 65.6 respectively) showed differences in the shape of their stability profiles [35]. However, irrespective of the genome GC-content a low stability peak was observed upstream of TLS in all systems. Detailed analysis of DNA structural property profiles (stability, bendability and curvature) for the above mentioned three systems revealed that stability could delineate promoter regions better than other properties (can be inferred from the Z-score plots for *E. coli, B. subtilis *and *M. tuberculosis *shown in PromBase) [46]. Thus PromBase can help the research community to compare and analyze the features present in the vicinity of TLS of various microbial genomes, from different phyla or with different GC-content. Cumulative CG-skew and TA-skew (skew^c^) plotted for whole genome sequences can be used to determine the position of the origin of replication in bacterial species [47]. The shape of the CDS skew shown in PromBase for all microbial genomes could be helpful in observing the specific trends followed for the gene orientation throughout the genome, which has been suggested to be the main factor responsible for the observed nucleotide skews [38,48].

### Statistics of genome characteristics which are displayed in PromBase

**Figure 4:**
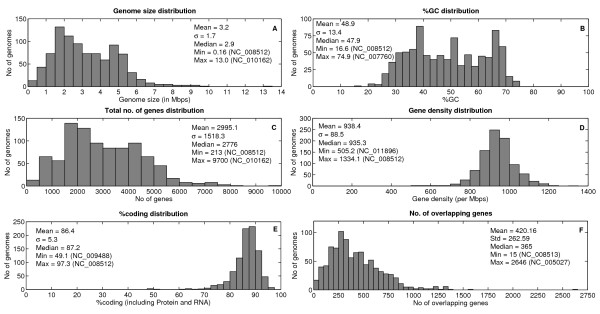
**General feature distribution in 913 microbial genomes**. The statistics for each of the features is given alongside the histograms. A) genome size B) %GC content C) Total number of genes D) Gene density E) %coding region (including protein and RNA) F) Number of overlapping genes.

As there is rapid accumulation of bacterial genomes over a decade, prokaryotic genomes have been analyzed for their genomic features and their variation among phyla [1,49-51]. It is important to analyze the general genome characteristics (particularly the GC-content distribution) of all bacterial genomes for a better evaluation of prediction results from any method. Hence we have carried out a comprehensive and quantitative analysis of the genome features such as, genome size, %GC-content, total number of genes, gene density and %coding region for all 913 microbial genomes downloaded from NCBI. Figure 4 illustrates the statistics of the above mentioned features. A large number of genomes have ~2 Mbp and ~5 Mbp genome size (246 genomes within 1.5 to 2.5 bins and 161 genomes within 4.5 to 5.5 bins respectively in Figure 4A). *Sorangium cellulosum 'So ce 56' *(NC_010162) from Delta-proteobacteria phylum is the largest genome (13 Mbp) with highest number of genes (9700). *Candidatus Carsonella ruddii PV *(NC_008512) which belongs to Gamma-proteobacteria phylum is the smallest genome (0.16 Mbp), has lowest %GC content (16.6%) and least number of genes (213). But it has highest gene density (1334 nucleotides per Mbps) and highest amount of %coding region (97.3%). The base composition of bacteria varies extensively between species. *Anaeromyxobacter dehalogenans 2CP-C *(NC_007760) genome (belonging to Delta-proteobacteria) has highest amount of GC (74.9%) which has been attributed to mutational bias [52,53] while a maximum number of genomes (144 and 145) have their GC distribution within the range of 35-40% and 65-70% respectively (Figure 4B). The percentage of coding sequence in a genome is very much higher in prokaryotes as compared to eukaryotes. On an average 86.4% bacterial genome sequence is coding either for protein or for RNA. The %coding region for the genomes *Orientia tsutsugamushi str. Boryong *(NC_009488; Phylum: Alphaproteobacteria), *Mycobacterium leprae Br4923 *and *TN *(NC_011896 and NC_002677; Phylum: Actinobacteria) is less as compared to the %intergenic region in the respective genomes.

The number of overlapping genes is also high in prokaryotes due to the dense packing of genetic elements. Figure 4F shows the overlapping gene distribution in all microbial genomes. Since there is a tail towards the higher end, it leads to the mean value being higher (420 genes) and it is more meaningful to consider the median value (365 genes). It has been found that the increase in overlapping genes is caused more frequently by mutations at 3'-end of gene in closely related species and mutations at 5'-end of gene in distant species [54]. Another study had revealed that a large number of misannotations happened at 5'-end due to mispredictions of start codons among co-directional and divergently oriented genes [55] and a database has been developed to analyze the reliability of overlapping gene structures [56]. PromBase also provides a list of overlapping genes with a download option.

### Distribution of TAN, DIV, CON intergenic and CODING regions within microbial genomes and the conservation of their length and %GC distribution

**Figure 5:**
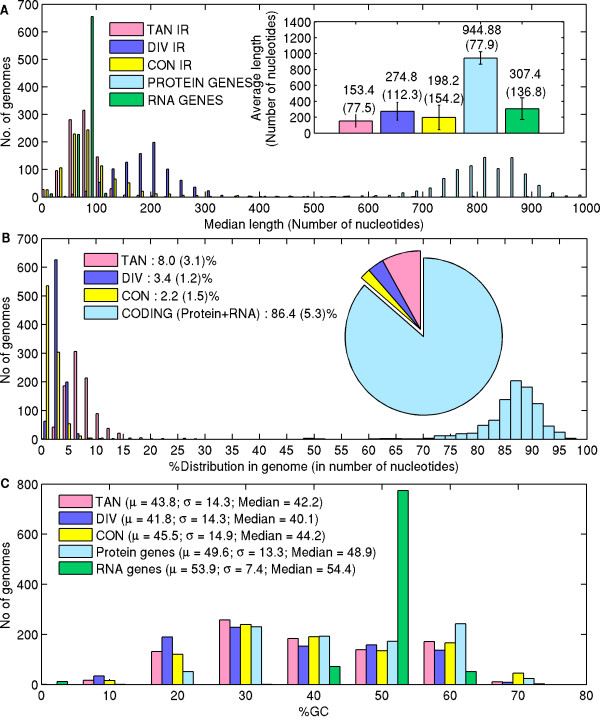
**Analysis of TAN, DIV, CON intergenic regions and CODING regions**. A) Length distribution of different regions in bacterial genomes. Inner bar chart shows the average length of different regions in all 913 bacterial genomes. The standard deviation for length of different regions is indicated as error bar (values are given in brackets). B) Percentage distribution of different intergenic and coding regions in bacterial genomes. Inner pie chart gives the overall average distribution in all bacterial genomes. The average value and the standard deviations (in bracket) are shown with the legend. RNA gene distribution is very small (0.73%) as compared to others, hence it is not shown in the figure. C) GC-content distribution in different regions of bacterial genomes. The overall mean, standard deviation and median values are also given.

The prokaryotic genomes consist largely of proteins genes and structural RNAs and only a small fraction constitutes the non-coding DNA. With the increase in genome sequence assembly of bacterial genomes, we have repeated the statistical analysis of the various types of intergenic regions in the bacterial genomes and the results are shown in PromBase for each genome. Figure 5 shows the conservation of relative lengths distribution and %GC-content of TANDEM (TAN), DIVERGENT (DIV), CONVERGENT (CON) and CODING regions in all 913 bacterial genomes. In general, the trend of protein coding genes > RNA genes > DIV IR regions > CON IR regions > TAN IR regions (Figure 5A) is shown to be retained. The length of these intergenic spacers between genes are thought to be important since they are the sites for regulatory signals [57]. The average length of divergent intergenic region being longer as compared to the other intergenic regions might be essential as they contain upstream regulatory signals for two genes. The convergent gene length distribution has a tail for long CON IR, which is reflected in the standard deviation value (154.2), being almost equal to the mean (198.2). The DIV IR length distribution also has an extended tail towards the higher values, but the distribution is broader as compared to the CON IR length distribution, as showed earlier for 39 bacterial genomes [57]. Though the average length of TAN IR region is smaller it has higher spread than compared to other types of intergenic regions in microbial genomes (Figure 5B). This suggests that the number of genes that are transcribed together or the chances for the adjacent genes to form a gene cluster or an operon is high in bacterial genomes [58-61], in order to optimize the energy expenditure for the expression of highly expressed genes under specific growth conditions. Four microbial genomes have ~50% genome comprising the intergenic region (*Orientia tsutsugamushi str. Boryong, Mycobacterium leprae Br4923, Mycobacterium leprae TN, Sodalis glossinidius str. 'morsitans'*). Comparative genome studies have revealed a drastic gene reduction and decay in *Mycobacterium leprae *genome by retaining only a minimal set of genes (less than half of the genome contain functional genes, while having abundant amount of *pseudogenes*) [62]. Analysis on accumulation of *pseudogenes *in *Mycobacterium leprae *genome has revealed the functional relevance of gene order within operons [63]. This study indicated that functionally less important genes have tendency to be located at the end of the operons, while more relevant genes tend to be located towards operon start. This particular genome has been shown as an extreme example for reductive evolution, the process by which large scale loss of gene function arises by inactivating the genes once their functions are no longer required in the highly specialized niches [64].

Figure 5C shows the %GC-content distribution in all four regions. It is clearly seen that the regional rule, RNA gene %GC > Protein gene %GC > CON IR %GC > TAN IR %GC > DIV IR %GC which has been reported earlier for 183 genomes [65] is maintained, with 689 bacterial genomes satisfying the general regional rule. Among the remaining 224 genomes, 81 did not satisfy the first rule (i.e. %GC coding > %GC CON IR), 105 did not follow the second criteria (i.e. %GC CON IR > %GC TAN IR) and 22 did not follow the last rule (i.e. %GC TAN IR > %GC DIV IR). For 16 genomes two rules are not satisfied. The regional rule applies to a huge number of bacterial genomes and it has been suggested that it is correlated with DNA structural properties such as stability, bendability and curvature [65].

Thus PromBase gives an overview of a large number of nucleotide composition as well as structural properties for every microbial genome. There are a few other databases available for viewing genomic properties wherein the results are presented graphically as radial plots or 'atlas' and as a Z score plot for each structural property [66-68]. However, these databases do not analyze the characteristic features of different intergenic and coding regions in detail, as presented in PromBase, hence combining the information from these databases with PromBase will allow for a better understanding of genome information content.

## Conclusion

PromBase can serve as a user friendly single point resource for the microbial genomic community to access information about several important genomic features for the genome of their interest. It helps to visualize the features present in the vicinity of TLS of various microbial genomes from different phyla or with different GC-content and also to explore the annotation of putative promoter regions, which could aid in transcriptional regulation of a gene. The web interface of PromBase is well organized and it also provides a download option for whole genome annotation of promoter regions. The free energy based classification scheme followed for categorizing the reliability of the predicted regions could help the experimentalists in designing their experiments. In addition to providing users a friendly input/output interface, PromBase gives a genome browser view for the annotated promoter regions in whole genome.

## Availability and requirements

Project Name: PromBase: A web resource for various genomic features and predicted promoters in prokaryotic genomes.

Project home page: http://nucleix.mbu.iisc.ernet.in/prombase/

User side requirements: Any standard WWW browsers, such as firefox and internet explorer

Server side requirements:

Operating system: Linux.

Programming and scripting languages: HTML, MySQL, PERL, MATLAB

This database is freely accessible for all academic and non-academic users.

## List of abbreviations

AFE: Average free energy; TSSs: Transcription start sites; TLSs: Translation start sites; CDS: Coding sequence.

## Competing interests

The authors declare that they have no competing interests.

## Authors' contributions

VR analyzed the data for general genome features displayed in PromBase, developed the database and wrote the manuscript. MB designed and supervised the study, corrected the manuscript and provided comments on the website construction and manuscript organization. All authors read and approved the final manuscript.

## Supplementary Material

Additional file 1 **Method details**. Contains the details of the methods followed for the calculation of each feature presented in PromBase. Click here for file

Additional file 2 **Figure S1 and Figure S2**. Figure S1-Relational database schema used to construct PromBase; **Figure S2 **- PromBase results page for tabulation of promoter prediction results along with the gene table that lies within the variable size window of the genome browser view. Click here for file
