# Supplementary material for: PromBase: a web resource for various genomic features and predicted promoters in prokaryotic genomes
Source: BMC Res Notes. 2011 Jul 22;4:257. doi: 10.1186/1756-0500-4-257 (PMC3160392; doi:10.1186/1756-0500-4-257)
Supplement: Additional file 1 — Method details. Contains the details of the methods followed for the calculation of each feature presented in PromBase. [file 1756-0500-4-257-S1.PDF]

Methods

Stability (average free energy) calculation

The stability of a double stranded DNA molecule can be expressed in terms of the free energy of its constituent base paired dinucleotides. The free energy change (ΔG^o_{37}) corresponding to the melting transition of an 'n' nucleotide (or 'n-1' dinucleotides) long DNA molecule, from double strand to single strand is calculated as follows [1]:

$$\Delta G^o = -\left(\Delta G^o_{ini} + \Delta G^o_{sym}\right) + \sum_{i=1}^{n-1} \Delta G^o_{i,i+1}$$

where,

ΔG^o_{ini} is the initiation free energy for dinucleotide of type ij.

ΔG^o_{sym} equals +0.43 kcal/mol and is applicable if the duplex is self-complementary.

$\Delta G^o_{i,j}$ is the standard free energy change for the dinucleotide of type ij.

The two terms ΔG^o_{ini} and ΔG^o_{sym}, which are more relevant for oligonucleotides, are not considered since our analysis involves long continuous stretches of DNA molecules. In the present study free energy over this long continuous stretch of DNA sequence was calculated by dividing the sequence into overlapping windows of 15 base pairs (or 14 dinucleotide steps). For each window, the free energy is calculated as given in the above equation and the free energy value is assigned to the central base pair of the window [2]. The energy values corresponding to the 10 unique dinucleotide sequences are taken from the unified parameters obtained from melting studies on 108 oligonucleotides [1, 3].

Curvature calculation

Curvature for DNA sequences has been calculated using in-house software NUCGEN [4]. Dinucleotide parameters based on crystal structure data of oligonucleotides (CS model) [5] and on relative gel mobility data (BHMT model) [6] have been used to calculate the curvature. For a promoter sequence of length 'n' and with a window size 'w' = 75 bp, curvature has been obtained for ($n - w + 1$) number of DNA fragments. Ratio of end-to-end distance 'd' to the contour length 'l_{max}' along the path traced by the DNA molecule (d/l_{max}) has been plotted as curvature profile with respect to the nucleotide position [7].

Bendability calculation

Bendability has been calculated using two tri nucleotide models, DNase I sensitivity [8] and Nucleosomal positioning preference [9]. The bendability profiles are calculated by looking up the values of trinucleotide parameters corresponding to each consecutive overlapping trinucleotides in the sequence [7, 10]. The bendability profiles were smoothened over a 30nt window.

Z score calculation

Z score has been calculated for each of the DNA structural property profile and plotted against the nucleotide position of the DNA sequence. The Z-score associated with the ith observation of a random variable X is given by

$$Z_i = \frac{X_i - \mu}{\sigma}$$

where, μ is the mean and σ is the standard deviation of all observations X_1, X_2…..X_n.

The quantity Z represents the distance between the raw score and the population mean in units of the standard deviation. Z is negative when the raw score is below the mean, positive when above.

CG and TA-skew

CG-skew and TA-skew, which has been used to determine the position of the origin of replication in bacterial species [11], was calculated using the following formulae [12, 13],

$$CG - skew = \frac{(C - G)}{(G + C)}$$

$$TA - skew = \frac{(T - A)}{(A + T)}$$

A sliding window (w) of size 1000nt with 1nt shift has been used to calculate the skews along the whole genome sequences. In order to avoid the dependence on window size and chromosome length (c), the skew values were normalized by multiplying with w/c [14]. Cumulative CG-skew and TA-skew ($skew^c$) has been calculated and plotted against the nucleotide position along the respective chromosome of microbial genomes.

The TA and CG strand asymmetries were reported in the vicinity of transcription start sites (TSS) of plants, fungi and human genomes [12, 15]. Hence, in PromBase the plot for calculated TA and CG skew values for 1001nt long sequences (spanning -500 to +500) aligned with respect to the TLS of protein genes were also displayed for all microbial genomes. The skew values were calculated using a sliding window of 100nt.

CDS-skew

Cumulative CDS-skew (CDS-skew^c) illustrates the bias, if any, in the directionality of the genes present along the chromosome length [16]. If a gene is transcribed in the forward strand of the chromosome, then a value of +1 is added to the cumulative skew and if it is transcribed in the reverse strand then it is subtracted from the cumulative skew (corresponds to a value of -1). The CDS-skew^c is plotted along the genome position. The space increment covered in each step along X-axis is equal to the gene length.

Promoter prediction methodology

The stability of DNA sequence has been calculated as described above. The scoring function defined below has been used to calculate the relative stability (DE) between neighboring regions of 100nt length with respect to every nucleotide position n. The average energy was assigned to the centre position corresponding to 'n + 50'.

$$DE_{(n+50)} = E1_{(n+50)} - E2_{(n+50)}$$

where,

$$E1_{(n+50)} = \frac{\sum_{n}^{n+100} \Delta G^{\circ}}{100}$$

$$E2_{(n+50)} = \frac{\sum_{n+150}^{n+250} \Delta G^{\circ}}{100}$$

Thus $E1_{(n + 50)}$ and $E2_{(n + 50)}$ represent the free energy averages for 100nt fragments starting from nucleotides 'n' and 'n + 150', respectively. DE is the difference between E1 and E2. A stretch of DNA sequence is assigned as a promoter only if its average free energy (E1) and the difference in free energy (DE) as compared to its neighbouring downstream region are greater than the chosen threshold values (E and D) for the corresponding %GC range. The threshold values have been calculated for genomic DNA with varying GC-content and have been applied to annotate

for promoter regions in all microbial genome sequences [17].

References

1. SantaLucia J, Jr.: **A unified view of polymer, dumbbell, and oligonucleotide DNA nearest-neighbor thermodynamics.** *Proc Natl Acad Sci U S A* 1998, **95:**1460-1465.
2. Kanhere A, Bansal M: **A novel method for prokaryotic promoter prediction based on DNA stability.** *BMC Bioinformatics* 2005, **6:**1.
3. Allawi HT, SantaLucia J, Jr.: **Thermodynamics and NMR of internal G.T mismatches in DNA.** *Biochemistry* 1997, **36:**10581-10594.
4. Bansal M, Bhattacharyya D, Ravi B: **NUPARM and NUCGEN: software for analysis and generation of sequence dependent nucleic acid structures.** *Comput Appl Biosci* 1995, **11:**281-287.
5. Bansal M: **Structural variations observed in DNA crystal structures and their implications for protein-DNA interactions.** *in Biological structure and Dynamics, Proceedings of the Ninth Conversation* 1996, **I:**121-134.
6. Bolshoy A, McNamara P, Harrington RE, Trifonov EN: **Curved DNA without A-A: experimental estimation of all 16 DNA wedge angles.** *Proc Natl Acad Sci U S A* 1991, **88:**2312-2316.
7. Kanhere A, Bansal M: **Structural properties of promoters: similarities and differences between prokaryotes and eukaryotes.** *Nucleic Acids Res* 2005, **33:**3165-3175.
8. Brukner I, Sanchez R, Suck D, Pongor S: **Trinucleotide models for DNA bending propensity: comparison of models based on DNaseI digestion and nucleosome packaging data.** *J Biomol Struct Dyn* 1995, **13:**309-317.
9. Satchwell SC, Drew HR, Travers AA: **Sequence periodicities in chicken nucleosome core DNA.** *J Mol Biol* 1986, **191:**659-675.
10. Pedersen AG, Baldi P, Chauvin Y, Brunak S: **DNA structure in human RNA polymerase II promoters.** *J Mol Biol* 1998, **281:**663-673.
11. Nikolaou C, Almirantis Y: **A study on the correlation of nucleotide skews and the positioning of the origin of replication: different modes of replication in bacterial species.** *Nucleic Acids Res* 2005, **33:**6816-6822.
12. Fujimori S, Washio T, Tomita M: **GC-compositional strand bias around transcription start sites in plants and fungi.** *BMC Genomics* 2005, **6:**26.
13. Lobry JR: **Asymmetric substitution patterns in the two DNA strands of bacteria.** *Mol Biol Evol* 1996, **13:**660-665.
14. Grigoriev A: **Analyzing genomes with cumulative skew diagrams.** *Nucleic Acids Res* 1998, **26:**2286-2290.
15. Touchon M, Nicolay S, Arneodo A, d'Aubenton-Carafa Y, Thermes C: **Transcription-coupled TA and GC strand asymmetries in the human genome.** *FEBS Lett* 2003, **555:**579-582.
16. Touchon M, Rocha EP: **From GC skews to wavelets: a gentle guide to the analysis of compositional asymmetries in genomic data.** *Biochimie* 2008, **90:**648-659.
17. Rangannan V, Bansal M: **High Quality Annotation of Promoter Regions for 913 Bacterial Genomes.** *Bioinformatics* 2010, **26:**3043-3050.
