# Supplementary material for: PromBase: a web resource for various genomic features and predicted promoters in prokaryotic genomes
Source: BMC Res Notes. 2011 Jul 22;4:257. doi: 10.1186/1756-0500-4-257 (PMC3160392; doi:10.1186/1756-0500-4-257)

Supplementary figures

Figure S1. Relational database schema used to construct PromBase. Main relationship between tables and the queries performed are shown. Each box represents a conceptual representation of the structured data. Description about each table is given at top. PK - primary key; FK - foreign key. Arrows indicate the relation of an entity to a reference.

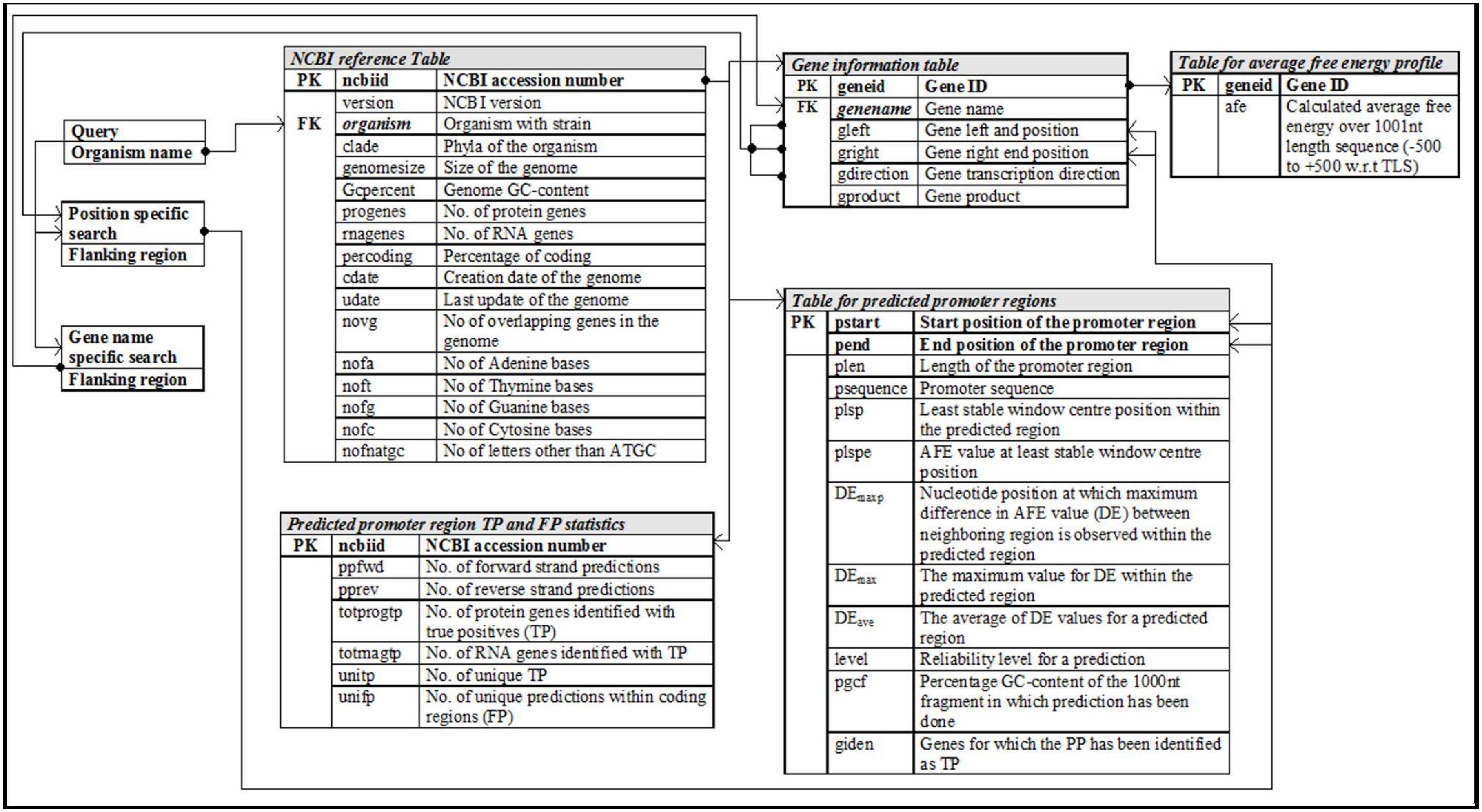

Figure S2. PromBase results page for tabulation of promoter prediction results along with the gene table that lies within the variable size window of the genome browser view. Average free energy profile for a region spanning -500 to +500 w.r.t TLS of each gene that is tabulated can be accessed using the hyper link provided for GenBank gene ID of respective gene. The block arrow indicates the page transition caused by clicking the hyperlink.

PromBase RESULTS

Genes within the region: 70000 to 74000 nts

S.No.	Gene ID	Gene Name	Gene left end position	Gene right end position	Length	Transcription direction	Gene product
1	b0063	araB*	68348	70048	1701	-	PROTEIN:L-ribulokinase
2	b0064	araC	70387	71265	879	+	PROTEIN:DNA-binding transcriptional dual regulator
3	b0065	yabI*	71351	72115	765	+	PROTEIN:conserved inner membrane protein
4	b0066	thiQ*	72229	72927	699	-	PROTEIN:thiamin transporter subunit
5	b0067	thiP*	72911	74521	1611	-	PROTEIN:fused thiamin transporter subunits of ABC

*indicates genes for which TPs are identified
Click on the Gene ID to get the free energy profile over -500 to +500nt region w.r.t TLS of the respective gene

Predicted Promoters within the region: 70000 to 74000 nts with trascription direction as ' + '

S.No.	Start	End	Length	Sequence	Least stable window centre	D_{max} position	Gene associated with TP	Prediction Reliability
1	70520	70533	14	atattctCaatctc	70525	70527	NA	Medium
2	71267	71285	19	tggtaacgaatcagacaaT	71270	71285	yabI	Medium
3	71308	71357	50	Agggtttgcagaatccctgcttcgtccatttgacaggcacattatgcaag	71354	71308	yabI	High
4	71602	71665	64	gttgcatcGctggtcatttctgaagaaaaacaaagcactacttgataaaactgaacatgc gttg	71646	71610	NA	Medium
5	72917	72964	48	agttttaacatttcgccccggtagtTtttcaatcacggtaaacagcag	72922	72942	NA	Medium
6	73294	73324	31	aaatcacaatgccgtcAgcagattgtggcag	73297	73310	NA	Medium

Least stable window centre is shown as lowercase bold letter and the D_{max} position is shown as uppercase bold letter in the promoter sequence
NA - Not assigned

Predicted Promoters within the region: 70000 to 74000 nts with trascription direction as ' - '

S.No.	Start	End	Length	Sequence	Least stable window centre	D_{max} position	Gene associated with TP	Prediction Reliability
1	70185	70080	106	acattgattatttgcacggcgtcacactTtgctatgccatagcatttttatccataagat tagcggatcctacctgacgctttttatcgcaactctctactgtttc	70131	70108	araB	High
2	71276	71255	22	atTcgttaccaattatgacaac	71260	71257	NA	Medium
3	71663	71600	64	aacgcatgttcagttttatcaagtagtgctttgtTttcttcagaaatgaccagcgatgc aacg	71645	71634	NA	Very high
4	72962	72894	69	tgctgtttaccgtgattgaaaaactaccggggcgaaatgttaaaacTgactgatatcacc tggctttac	72922	72940	thiQ	High
5	73322	73292	31	tgccacaaTctgctgacggcattgtgatttt	73296	73300	thiQ	Medium

Least stable window centre is shown as lowercase bold letter and the D_{max} position is shown as uppercase bold letter in the promoter sequence
NA - Not assigned

Promoter structural property profile

Escherichia coli str. K-12 substr. MG1655 (NC_000913)

Gene ID : b0063
Gene name : araB
TLS position: 70048
Gene length : 1701 nts
Direction of transcription : -
Gene Product : PROTEIN - L-ribulokinase

Average free energy profile

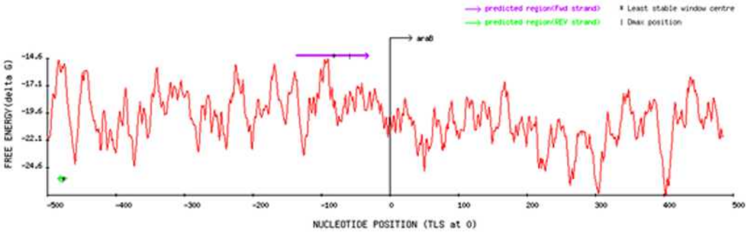

Supplement: Additional file 2 — Figure S1 and Figure S2. Figure S1-Relational database schema used to construct PromBase; Figure S2 - PromBase results page for tabulation of promoter prediction results along with the gene table that lies within the variable size window of the genome browser view. [file 1756-0500-4-257-S2.PDF]
